# Advanced Management of Hearing Loss: A Comprehensive Review of the Special Issue

**DOI:** 10.3390/jcm13237409

**Published:** 2024-12-05

**Authors:** Shuna Li, Ling Lu, Jun Yang, Maoli Duan

**Affiliations:** 1 Department of Otorhinolaryngology-Head & Neck Surgery, Xinhua Hospital Affiliated to Shanghai Jiaotong University School of Medicine, Shanghai 200082, China; lishuna@xinhuamed.com.cn; 2 Institute of Otology, School of Medicine, Shanghai Jiaotong University, Shanghai 200052, China; 3 Shanghai Key Laboratory of Ear and Nose Disease Transformation, Shanghai 200092, China; 4 Department of Otolaryngology-Head and Neck Surgery, Zhongda Hospital, Southeast University, Nanjing 210009, China; entluling60@126.com; 5 Department of Otolaryngology-Head & Neck, Audiology and Neurotology, Karolinska University Hospital, 171 76 Stockholm, Sweden; 6 Department of Clinical Science, Intervention and Technology, Division of Ear, Nose and Throat Diseases, Karolinska Institute, 171 77 Stockholm, Sweden

Hearing loss, affecting over 466 million people worldwide, poses significant challenges for affected individuals, their families, and healthcare providers alike [1]. The impact of hearing loss extends beyond communication difficulties, affecting mental health, social participation, and cognitive function. Recent advancements in genetics, audiology, and technology have revolutionized the management of hearing loss, offering new hope for those affected. Understanding the causes of hearing loss is crucial for developing targeted treatments. Conductive hearing loss is often caused by external and/or middle ear abnormalities, while sensorineural hearing loss results from damage to the inner ear or auditory nerve, which can be caused by noise exposure, aging, genetic factors, ototoxic drugs, etc. Central auditory processing disorders involve the central nervous system’s inability to process auditory information correctly [2].

The identification of genetic mutations responsible for hereditary hearing loss has led to the exploration of gene therapy and CRISPR-Cas9 technology to correct or mitigate the effects of these mutations [3,4]. Stem cell therapy and the development of small molecules to regenerate cochlear hair cells have promising potential for the treatment of sensorineural hearing loss. The efficacy of stem-cell therapy was once controversial. Recent studies have improved our understanding of the developmental pathways responsible for the generation of hair cells and spiral ganglion neurons. However, significant challenges remain in elucidating the molecular interactions required for stem cell differentiation and function as ear sensory cells. Some of the challenges of conventional stem cell therapies can be addressed by organoids [5]. More recently, AAV-OTOF gene therapy has revolutionized the field of treating OTOF-induced hearing problems such as auditory neuropathy. The first clinical application using AAV-OTOF gene therapy for patients with auditory neuropathy was investigated in China, and the results reported by Dr Chai’s team from Southeast University, Nanjing, have proven gene therapy to be an excellent option for treating OTOF-induced hearing loss without exerting heavy ototoxic and other toxic effects on the patients [6,7]. Thus, gene therapy has improved our understating regarding patients with sensorineural hearing loss in the field. However, further multicenter trials are needed in the near future.

Implantable auditory devices are constantly becoming smaller, less visible, and more efficient. With the development of technology, implantable hearing devices that can adjust themselves according to a patient’s hearing level and environment will further benefit patients [8]. Telehealth has been used by otologists and audiologists as a mode to deliver health care services. The integration of telehealth in audiology has expanded access to hearing care, particularly for individuals in remote areas or with limited mobility. Telehealth services emerged in response to the need for safe healthcare precipitated by the COVID-19 pandemic [9,10]. The development of small-molecule drugs and targeted drugs and the innovation of new drug delivery systems have provided good news to patients with different causes of hearing loss [11,12]. However, it may take some time to apply the above techniques to patients. We hope these newly developed techniques will soon be applied in clinical practice.

This Special Issue of the *Journal of Clinical Medicine* entitled “Clinical Management of Hearing Loss” contains six articles and one systematic review. The spectrum of research presented in this Special Issue spans from etiological studies to therapeutic interventions. These contributions shed light on the genetic underpinnings of hearing loss, advancements in surgical management, the role of pharmacological treatments, and the potential of gene/stem cell therapies. Notably, the findings reported in these studies significantly improve our understanding of unilateral sensorineural hearing loss, the association between viral infections and sudden hearing loss, and the impact of inflammatory factors on inner-ear function. The contributions made by the six articles and one review included in this Special Issue are summarized below:

1. A retrospective analysis showed the detection rates of otosclerosis in high-resolution computed tomography (HRCT) images of temporal bone obtained by general radiologists and the impact of incomplete radiological request forms on these rates. The results revealed that general radiologists detected otosclerosis at a significantly lower rate (36.1%) than subspecialist neuroradiologists (82.5%). In summary, this study reveals potential strategies for improving the accuracy of otosclerosis diagnosis, including refining radiology request processes, enhancing specialist training for radiologists, and strengthening communication between clinicians and radiologists.
2. The hearing rehabilitation characteristics of Mandarin-speaking cochlear implant (CI) patients were comprehensively analyzed. The results revealed a significant linear relationship between the aided hearing threshold (AHT) and speech perception accuracy (SPA). The timing of cochlear implantation and rehabilitation training significantly improved SPA, with early implantation and post-operative rehabilitation being crucial for patient recovery. This study underscores the importance of early intervention and tailored rehabilitation in order to enhance speech perception outcomes among CI patients.
3. The role of inflammatory factors, particularly IL-6, in inner-ear impairment among 146 recovered Omicron-infected patients was investigated. The findings revealed a significant correlation between IL-6 levels and inner-ear impairment, especially in the 18–60 age group. It was concluded that IL-6 is significantly associated with inner-ear impairment in Omicron-infected patients, highlighting the need for early hearing screening post-infection. This research underscores the importance of monitoring inflammatory markers in understanding and managing hearing impairments related to COVID-19 infections.
4. The clinical profiles and outcomes of adult patients with full-frequency idiopathic sudden sensorineural hearing loss (ISSNHL) undergoing combination therapy were investigated. The 131 patients received standardized treatment with intravenous methylprednisolone, batroxobin, and Ginkgo biloba extract. The authors found an overall recovery rate of 57.3%. Independent predictors of hearing outcomes included accompanying vertigo and body mass index (BMI), with patients with a BMI ≥ 22.4 kg/m^2^ showing a better chance of recovering their hearing. This study suggests that combination therapy could be particularly beneficial for ISSNHL patients with a higher BMI, highlighting the importance of considering BMI in treatment strategies.
5. This study investigates the potential association between Epstein–Barr Virus (EBV) infection and sudden sensorineural hearing loss (SSNHL) in an East Asian population. The researchers enrolled patients with unexplained hearing loss and conducted serological testing for EBV-specific antibodies and EBV DNA in serum. The results revealed a negative association between hearing recovery and viral DNA PCR levels after steroid therapy, indicating that EBV might play a role in SSNHL. However, larger-scale studies are needed to confirm these findings and explain the underlying mechanisms.
6. This study presents the first national survey on unilateral sensorineural hearing loss (UHL) in Japan, examining etiology, severity, audiogram type, and device usage for children and adults. It involved 15,981 patients, a significant number of whom were pediatric. Sudden sensorineural hearing loss was the most common cause across all ages, and more than half of the patients had moderate hearing loss. Only 4.4% of the UHL patients used hearing devices, primarily conventional hearing aids. This study highlights the limited use of hearing aids and cochlear implants among UHL patients in Japan and suggests that many potential candidates for receiving interventions, such as cochlear implants, remain untreated.
7. This systematic review evaluates the effects of bone conduction devices (BCDs) on patients with unilateral conductive hearing loss (UCHL). Its authors included 21 studies that met the specific inclusion criteria, examining data on hearing thresholds, speech recognition, sound localization, and subjective questionnaire outcomes. The results indicate moderate improvements in hearing thresholds and significant enhancements in speech recognition for UCHL patients using BCDs. However, sound localization varied widely among individuals. Subjective questionnaires revealed an overall positive impact of BCDs on daily life, despite some unfavorable experiences. The authors conclude that BCDs offer credible benefits for UCHL patients and recommend a trial period with non-implantable BCDs before considering permanent implants.

The articles published in this Special Issue not only advance our understanding of hearing loss but also pave the way for future research. The findings have the potential to influence clinical practices, inspire new therapeutic approaches, and guide the development of novel hearing devices and technologies. As we have shown, the future need for continued research into early intervention, diagnosis, and personalized management strategies requires further big data analysis and multicenter studies.
